# Fibroblast Growth Factor 9 (FGF9) negatively regulates the early stage of chondrogenic differentiation

**DOI:** 10.1371/journal.pone.0241281

**Published:** 2021-02-02

**Authors:** Xiaoyue Zhang, Mengjia Weng, Zhenqi Chen

**Affiliations:** 1 Department of Orthodontics, The Affiliated Stomatology Hospital of Tongji University, Shanghai, China; 2 Shanghai Key Laboratory of Stomatology, Shanghai, China; 3 Department of Orthodontics, Shanghai Ninth People’s Hospital, Shanghai Jiao Tong University School of Medicine, Shanghai, China; Duke University School of Medicine, UNITED STATES

## Abstract

Fibroblast growth factor signaling is essential for mammalian bone morphogenesis and growth, involving membranous ossification and endochondral ossification. FGF9 has been shown to be an important regulator of endochondral ossification; however, its role in the early differentiation of chondrocytes remains unknown. Therefore, in this study, we aimed to determine the role of FGF9 in the early differentiation of chondrogenesis. We found an increase in FGF9 expression during proliferating chondrocyte hypertrophy in the mouse growth plate. Silencing of FGF9 promotes the growth of ATDC5 cells and promotes insulin-induced differentiation of ATDC5 chondrocytes, which is due to increased cartilage matrix formation and type II collagen (col2a1) and X (col10a1), Acan, Ihh, Mmp13 gene expression. Then, we evaluated the effects of AKT, GSK-3β, and mTOR. Inhibition of FGF9 significantly inhibits phosphorylation of AKT and GSK-3β, but does not affected the activation of mTOR. Furthermore, phosphorylation of inhibited AKT and GSK-3β was compensated using the AKT activator SC79, and differentiation of ATDC5 cells was inhibited. In conclusion, our results indicate that FGF9 acts as an important regulator of early chondrogenesis partly through the AKT/GSK-3β pathway.

## Introduction

In mammals, endochondral ossification begins with the formation of cartilage from undifferentiated mesenchymal cells, involving multiple steps, including proliferation and hypertrophy of chondrocytes [1]. Proliferation and hypertrophy of chondrocytes together with extracellular matrix synthesis act as the major contributors to longitudinal growth of bone [2]. The production and maturation of chondrocytes is sequentially and tempo-spatially controlled by multitude of systemic and local factors, among which several growth factors and cytokines such as fibroblast growth factor (FGFs), as well as the intracellular signaling pathways play a key role [3].

Fibroblast growth factor 9 (FGF9) known as one of 22 members of the fibroblast growth factors, was initially obtained from supernatant of a human glioma-derived cell lines and used to nourish primary rat glial cells [4]. Mouse FGF9 contains three exons and two introns on chromosome 14, which encodes the protein sharing 99% amino acid sequence with human FGF9 [5]. Fibroblast growth factor signaling is essential for the mammalian skeleton morphogenesis and growth, involved in both membranous ossification and endochondral ossification [6]. FGF9 transcripts were detected in the limb apical ectodermal ridge of an E10.5 mouse embryo and cartilage primordia of the E12.5 mouse embryo forelimb [4, 7]. Mice lacking FGF9 exhibit a disproportionate shortening of proximal skeletal elements, a prominent defect observed in patients afflicted with FGFR3-induced chondrodysplasia syndromes [8].

Several studies have shown that downregulation of FGF9 signaling in mice inhibits the proliferation of chondrocytes and the differentiation of proliferative chondrocytes to hypertrophic chondrocytes [8–10]. In contrast, targeted overexpression of FGF9 to cartilage of transgenic mice reduced proliferation and terminal differentiation of chondrocytes in the growth plate [7]. During in vitro hMSC chondrogenic differentiation, FGF9 exhibited a negative effect when present throughout the entire differentiation program [11]. Furthermore, FGF9 promoted proliferation and had no effect on differentiation but inhibited terminal differentiation in a rat calvaria-derived cell line (RCJ 3.1C5.18) that spontaneously undergoes chondrocyte differentiation in vitro [12]. However, costal chondrocytes combined with exogenous FGF9 were able to simultaneously promote the chondrogenesis of dental pulp stem cells and partially inhibit their mineralization by enhancing the phosphorylation of ERK1/2 [13]. From the studies above, FGF9 participates in cartilage development, but the function of FGF9 in the chondrogenic differentiation has not been dissected.

In this study, we made use of chondroprogenitor ATDC5 cells to investigate the effects of FGF9 in early chondrogenic differentiation. ATDC5 cells, which originally isolated from the feeder-independent teratocarcinoma stem cell line AT805, were widely used because of its chondrogenic potential in the presence of insulin [14]. We silenced FGF9 in ATDC5 cells, detected the expression of chondrogenic genes at mRNA and protein levels, and performed cartilage-related staining. We also examined the phosphorylation of AKT, GSK-3β, mTOR to explore the underlying regulation mechanism of FGF9.

## Materials and methods

### Immunohistochemical staining

The animals used in this research were provided by the Ninth People’s Hospital Animal Center (Shanghai, China). All animal protocols were approved by the Animal Care and Experiment Committee of the Ninth People's Hospital.

The knee joints, temporomandibular joints and the cranial base synchondrosis of C57BL/6 mice were micro-dissected at postnatal day (P) 0.5 and P7, and fixed in 4% paraformaldehyde for 48h at 4°C. Then, the samples were sliced into 5-μm-thick sections after decalcification and embedding. The expression of FGF9 was evaluated following the standard protocols. Briefly, the deparaffinized sections were heat-treated with citrate buffer (pH 6.0) for 20 min to retrieve the antigens. The sections were incubated with 3% hydrogen peroxide for 15 min to block endogenous peroxidase, followed by 10% goat serum for 15 min to block tissue nonspecific-binding sites. After washing, the samples were then incubated with primary antibody for FGF9 (Abcam) overnight at 4°C (dilution 1:200). Subsequently, the samples were treated with an antibody amplifier and the HRP polymer for 10 min, respectively, followed by detection with the use of diaminobenzidine tetrahydrochloride. Finally, the samples were counterstained with hematoxylin, and examined under a microscope (Olympus).

### Cell culture and chondrogenic differentiation

The undifferentiated ATDC5 cells purchased from Cobioer Biosciences Co., Ltd., were cultured in Dulbecco’s modified Eagle’s medium/F12 (1:1) medium (Sigma) supplemented with 5% fetal bovine serum (Gibco). To induced chondrogenic differentiation, ATDC5 cells were seeded in 6-well plates or 12-well plates, and the original medium was replaced with the differentiation medium consisting of α-MEM medium (Sigma), 1% insulin-transferrin-selenium (ITS, Sigma) and 5% fetal bovine serum (Gibco) once the cells reached confluence. The differentiation medium was changed every other day for 15 days.

### FGF9 lentivirus transfection

FGF9-shRNA lentiviruses-mediated RNA interference was used to downregulate the gene expression of FGF9 in ATDC5 cells. The FGF9 target sequence was CCCUGACAAAGUACCUGAA and non-mammalian sequence was used as a control. 72h after transfection which followed the standard protocol, the cells were selected with puromycin at a concentration of 5 μg/ml for 10 days. The selected clones were then designated as ATDC5-FGF9-shRNA and ATDC5-control and cultured for subsequent experiments.

### MTT assay and BrdU assay for cell proliferation

On day 0, ATDC5-FGF9-shRNA and ATDC5-control cells were seeded in 96-well plates with 5000 cells and 100 μl cultural medium per well. Samples were collected from each group on day 1, 2, 3, 4, and 5, respectively. MTT and DMSO were used to monitor cell viability and the absorbance of each well was measured at 490nm. All the experiments were conducted under the guidance of the manufacturer’s instructions and repeated at least three times.

5-Bromo-2’-deoxy uridine (BrdU, BOSTER) was added to the culture medium at a final concentration of 10 μM and incubated for 2 hours, when ATDC5-FGF9-shRNA and ATDC5-control cells reached about 50% confluence. The cells were then fixed in 4% paraformaldehyde for 20 minutes, and treated with anti-BrdU antibody (BOSTER) (dilution 1:200) at 4°C overnight. Cy3-labelled secondary antibody (BOSTER) was used and nucleic acids was stained with DAPI. The percentage of positive cells in the total cells was observed and recorded with a conventional fluorescence microscope.

### Safaranin-O staining and Alcian blue staining

ATDC5, ATDC5-FGF9-shRNA and ATDC5-control cells were seeded in 12‐well plates at a density of 50 000 cell/well and cultured for 15 days with the differentiation medium. Cells were fixed in 4% paraformaldehyde for 20 minutes at 4°C, and stained with 1% Alcian blue 8GX (Sigma) or 0.1% Safranin-O (Sigma) overnight at 4°C. The stained cells were washed with phosphate-buffered saline (PBS) for three times, then photographed with a scanning camera.

### Quantitative real-time polymerase chain reaction (PCR)

TRIzol (TaKaRa) was used to extract total RNA from the cells according to the manufacturer’s instructions. RNA was reverse transcribed into cDNA using a PrimeScript RT kit with a gDNA eraser (TaKaRa). Quantitative PCRs were performed with LightCycler/ LightCycler 480 System (Roche Diagnostics) using TB Green Premix Ex Taq (Tli RNaseH Plus) (TaKaRa)as appropriate. The mRNA level of the target gene was normalized to that of glyceraldehyde-3-phosphate dehydrogenase (GAPDH) and the relative gene expression was calculated by 2^–ΔΔCT^ method. The primers used were as follows: GAPDH, 5′-TGACAATGAATACGGCTACAGCA-3′ (forward) and 5′-CTCCTGTTATTATGGGGGTCTGG-3′ (reverse); FGF9, 5’-GTGAAGGAACCTTACTTCTGTGGTG-3’ (forward) and 5’-GTCCTTGGGGTCTTCTACCTTTCTC-3’ (reverse); Col2, 5′-AAGTCACTGAACAACCAGATTGAGA-3′ (forward) and 5′-AAGTGCGAGCAGGGTTCTTG-3′ (reverse); Col10, 5′-TGCAATCATGGAGCTCACAGA-3′ (forward) and 5′-CAGAGGAGTAGAGGCCGTTTGA-3′ (reverse); Acan, 5’-GTGGGTGGTGAAGACGACAT-3’ (forward) and 5’-GATGGGCTTTGCTGTAAGGA-3’ (reverse); Ihh, 5’-GTCCTATGCTCCTCTCACAAG-3’ (forward) and 5’-GATGGAAGGTGCTCTCTTCTAG-3’ (reverse); Mmp13, 5’-TgTTTgCAgAgCACTACTTgAA-3’ (forward) and 5’-CAgTCACCTCTAAgCCAAAgAAA-3’ (reverse); FGFR3, 5’-GTCTGGGCTAAGGATGGTAC-3’ (forward) and 5’-ATCTTCGTGGGAGGCATTTAG-3’ (reverse).

### Western blot analysis

Cells from different experimental conditions were lysed with ice‐cold RIPA lysis buffer and protease inhibitors. A BCA protein assay kit was used to measure the protein concentration according to the manufacturer's instructions. SDS‐PAGE electrophoresis was carried out with 20 μg per lane of lysate protein and 10% or 6% polyacrylamide gels, followed by electrophoresis onto polyvinylidene difluoride (PVDF) membranes. After the transfer, the membranes were blocked with 5% dried skimmed milk in TBST buffer (Solarbio, PH 7.6). The PVDF membranes incubated with primary antibodies in TBST, which contained 5% dried skimmed milk overnight at 4°C. The membranes were washed in TBST buffer, and then incubated with the secondary antibody for 1h at RT. Immunoreactivity was detected with the ECL substrate (Thermo Fisher Scientific). Anti-FGF9 antibody was purchased from Abcam. Collagen II polyclonal antibody was from Invitrogen, Collagen X antibody was from Abcam Technology, MMP13 polyclonal antibody was from Bioworld Technology, and Aggrecan antibody was from Affinity Biosciences. Primary antibodies (total or phospho-) specific for AKT (or Protein Kinase B), Glycogen synthase kinase 3 (GSK3), mammalian Target of Rapamycin (mTOR) were from Cell Signaling Technology. GAPDH expression was analyzed as an internal control during the Western blot analyses.

### Statistical analysis

All experimental groups were repeated at least three times, and the results were reported as means± standard errors. A two-tailed Student’s t-test was used to assess the differences between the two groups and P < 0.05 was considered statistically significant.

## Results

### Expression pattern of FGF9 in growth cartilage

**Fig 1:**
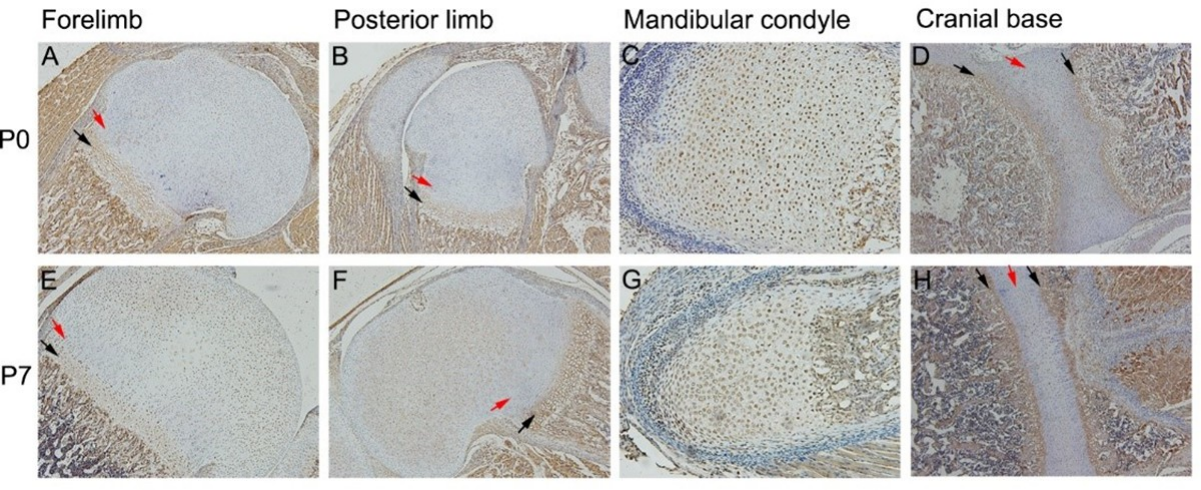
Immunolocalization of FGF9 in mouse limbs epiphyseal cartilage, mandibular condylar cartilage and cranial base synchondrosis. In P0 and P7 limbs epiphyseal cartilage and cranial base synchondrosis, positive staining of FGF9 in the hypertrophic chondrocytes and prehypertrophic chondrocytes (black arrow) was significantly higher than resting chondrocytes or columnar chondrocytes (red arrow) (A/B/D and E/F/H). No similar differences were found in mandibular condyle cartilage at P0 and P7 (C/G).

To investigate the expression patterns of FGF9 during chondrocytes differentiation in vivo, we analyzed cartilage specimens from the forelimbs, the posterior limbs, the mandibular condyle and the cranial base from P0 and P7 mice. At P0 and P7, the forelimb joints and the posterior limb joints developed well, and the maturity of chondrocytes showed a significant gradient. The differentiation process of prechondrogenic cells to terminally differentiated chondrocytes can be seen from the resting area to the hypertrophic area. FGF9 was detected throughout the epiphyseal chondrocyte differentiation of limb joints. Meanwhile, compared with resting chondrocytes or columnar chondrocytes, the expression of FGF9 was significantly increased in the hypertrophic chondrocytes and prehypertrophic chondrocytes (Fig 1A, 1B, 1E and 1F). This is consistent with the expression pattern of FGF9 in growth cartilage of the cranial base (Fig 1D and 1H). However, in the mandibular condyle cartilage specimens, the expression of FGF9 in columnar chondrocytes was increased compared with that in resting chondrocytes, and the change was minimal compared with the expression of FGF9 in the hypertrophic chondrocytes or prehypertrophic chondrocytes (Fig 1C and 1G).

### FGF9 expression increases during the differentiation of ATDC5 cells

**Fig 2:**
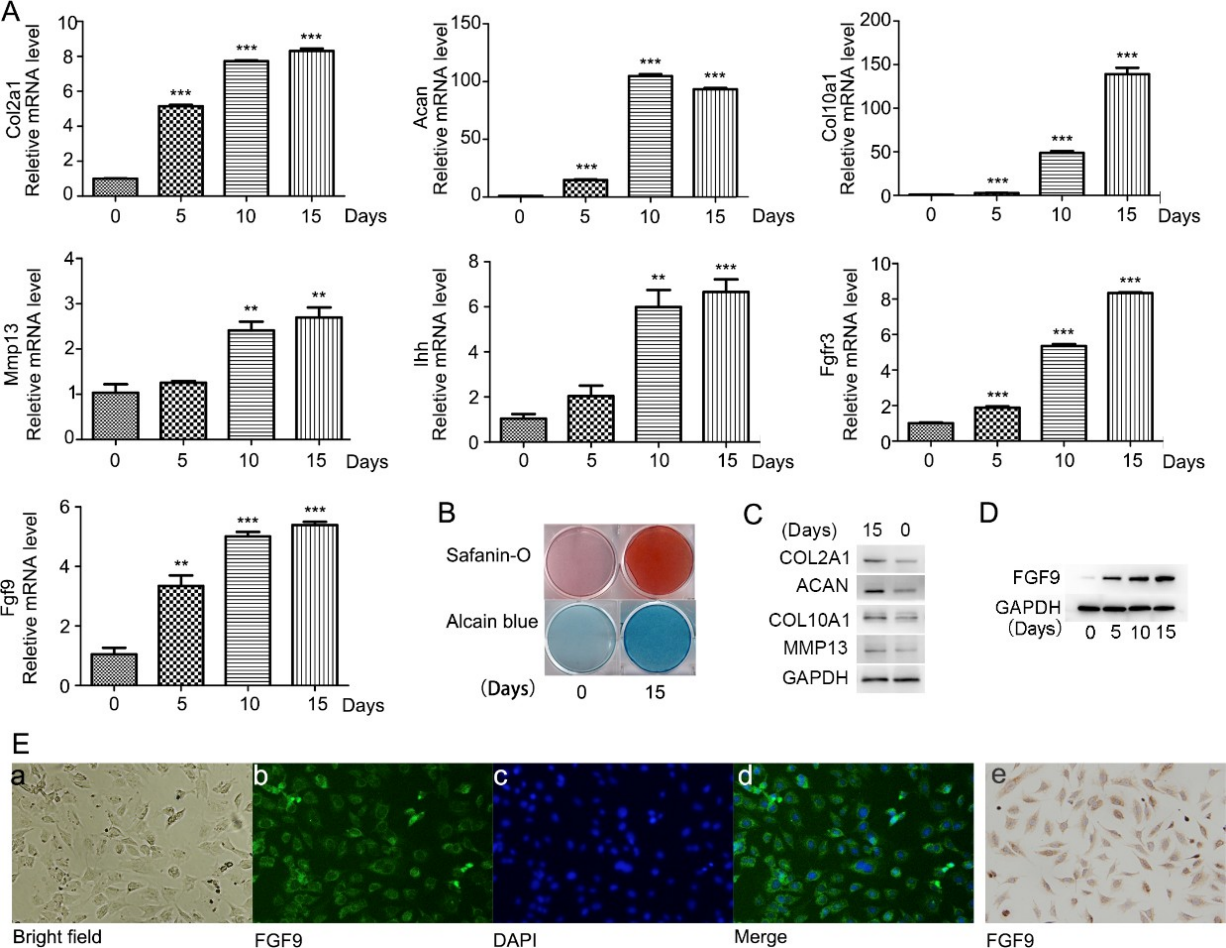
FGF9 expression increased during early differentiation of ATDC5 cells. ATDC5 cells effectively differentiated into chondrogenic cells and stayed at the early stage of chondrogenic differentiation by day 15 as detected by quantitative real-time PCR analysis of the chondrogenic genes Col2, Aggrecan, Col10, Ihh, Mmp13 and FGFR3 Safranin-O staining and Alcian blue staining were carried out on the 15th day and 0^th^ day of induction(B), as well as the protein levels of Col2a1, Acan, Col10a1, Mmp13 detected by Western blot (C). Quantitative real-time PCR (A) and Western blotting analysis (D) showed increased expression of FGF9 during 15 days of ATDC5 differentiation. Immunofluorescence staining (E/a-d) and immunohistochemical staining (E/e) were used to detect the expression and distribution of FGF9 in undifferentiated ATDC5 cells.

Immunofluorescence staining and immunohistochemical staining were performed for the identification of endogenous expression of FGF9, which indicated that FGF9 was mainly localized in the cytoplasm of undifferentiated ATDC5 cells (Fig 2E).When ATDC5 cells were induced to differentiate for 0, 5, 10, and 15 days, the mRNA expression of Col2a1 (collagen, type II, alpha 1), Acan (Aggrecan), Col10a1 (collagen, type X, alpha 1), Ihh (Indian hedgehog), Mmp13 (matrix metallopeptidase 13), and FGFR3 (fibroblast growth factor receptor 3) were detected. The chondrogenic gene expression was significantly increased during the induction (Fig 2A). The protein levels of Col2a1, Acan, Col10a1, Mmp13 increased after 15 days of induction (Fig 2C). Consistently, on the 15th day of insulin-induced ATDC5 cells differentiation, alcian blue staining and safranine O staining were carried out, which showed a significant increase in cartilage matrix deposition compared with that on the 0^th^ day (Fig 2G and 2H) (Fig 2B). All above results indicated that ATDC5 cells effectively differentiated into chondrogenic cells and stayed at the early stage of chondrogenic differentiation by day 15. During this process, mRNA expression and protein levels of FGF9 were significantly upregulated (Fig 2A and 2D).

### FGF9 silencing promotes the proliferation and chondrocyte differentiation of ATDC5 cells

**Fig 3:**
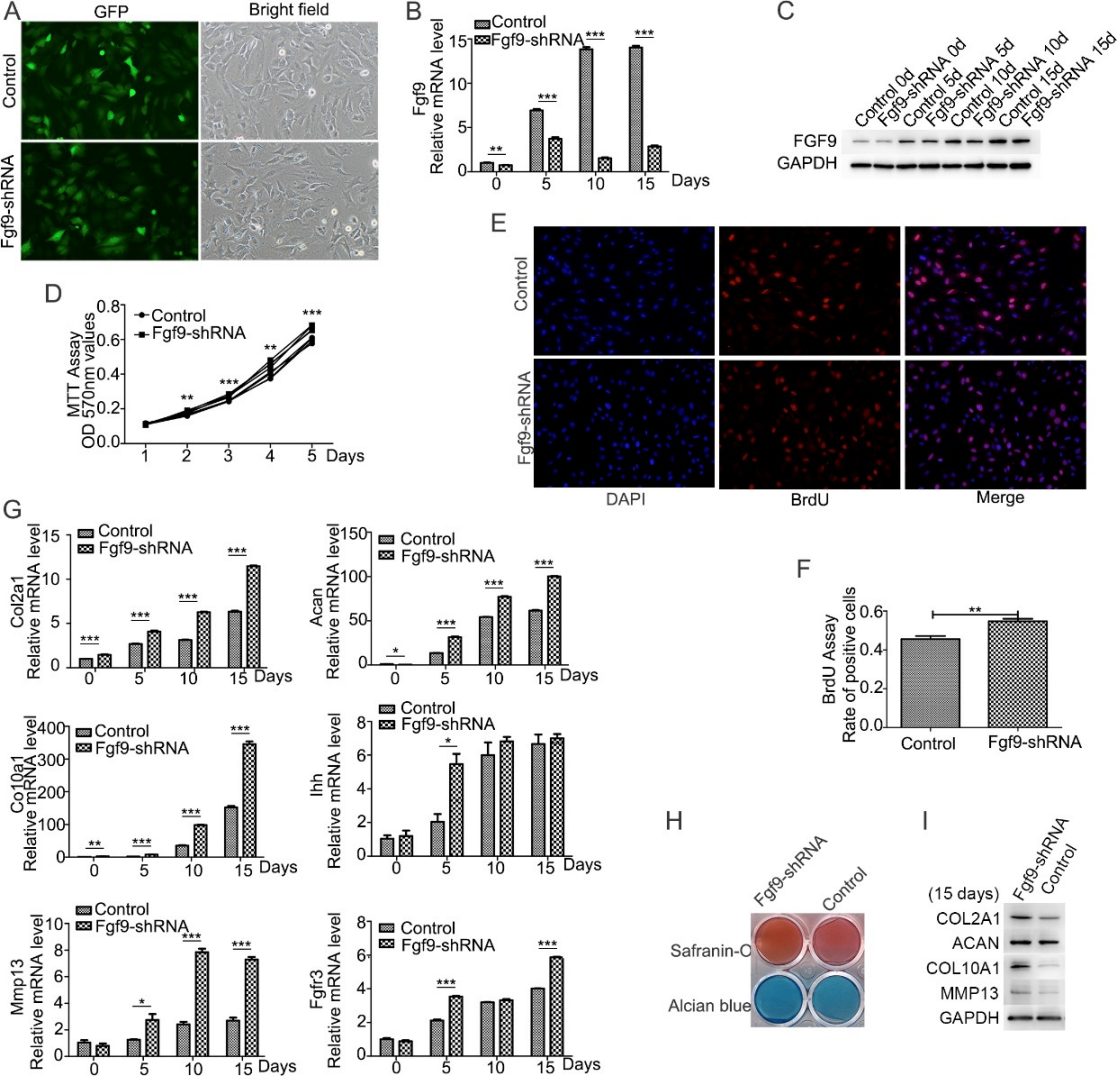
FGF9 silencing promoted the ITS-induced chondrogenic differentiation of ATDC5 cells. The efficiency of lentivirus-infected cells was observed by the same field fluorescence and bright light cell density under the control microscope (A). FGF9 silencing in the ATDC5-FGF9-shRNA cells was detected by quantitative real-time PCR (B) and Western blotting (C). MTT assay showed that FGF9 silencing promoted the proliferation of undifferentiated ATDC5 cells (D). Immunofluorescence staining was used to detect Brdu incorporation within 2 hours, Brdu positive cells were marked in red, and nuclear markers were blue (E), and the ratio of positive cells to total cells was calculated (F). Quantitative real-time PCR (G) and Western blotting (I) showed the increased expression of chondrogenic markers Col2a1, Acan, Col10a1, and Mmp13, when FGF9 was silenced. FGF9 silencing promoted Safranin-O staining and Alcian blue staining (E) on the 15th day of induction. (* P<0.05; **P<0.01; ***P<0.005).

FGF9 shRNA lentivirus was employed to transfect ATDC5 cells in order to investigate the effect of FGF9 on ATDC5 cells. The control group was ATDC5 cells transduced with scramble shRNA lentiviral particles. Comparison of GFP-positive and bright-fied images revealed that nearly 95% of cells were successfully infected with the lentivirus, and then stable clones were selected (Fig 3A). Compared to ATDC5 control cells, ATDC5-FGF9-shRNA cells showed downregulation of the mRNA expression and protein levels of FGF9 during the early chondrogenic differentiation (Fig 3B and 3C).

The effects of FGF9 on the proliferation of ATDC5 cells was evaluated with MTT assay and Brdu assay. MTT assay in 5 days showed that FGF9 silencing promoted the growth of ATDC5 cells in a time-dependent manner (Fig 3D). Immunofluorescence staining showed more Brdu-positive cells in ATDC5-FGF9-shRNA cell comparied with ATDC5-control cells (Fig 3E and 3F). Furthermore, silencing FGF9 upregulated mRNA expression and the protein levels of the chondrogenic markers Col2a1, Aan, Col10a1, and Mmp13 (Fig 3G and 3I), as well as cartilage matrix deposition compared to the controls (Fig 3H). It indicated that FGF9 silencing promoted the early chondrogenic differentiation.

### FGF9 affects the phosphorylation of AKT and GSK-3β

**Fig 4:**
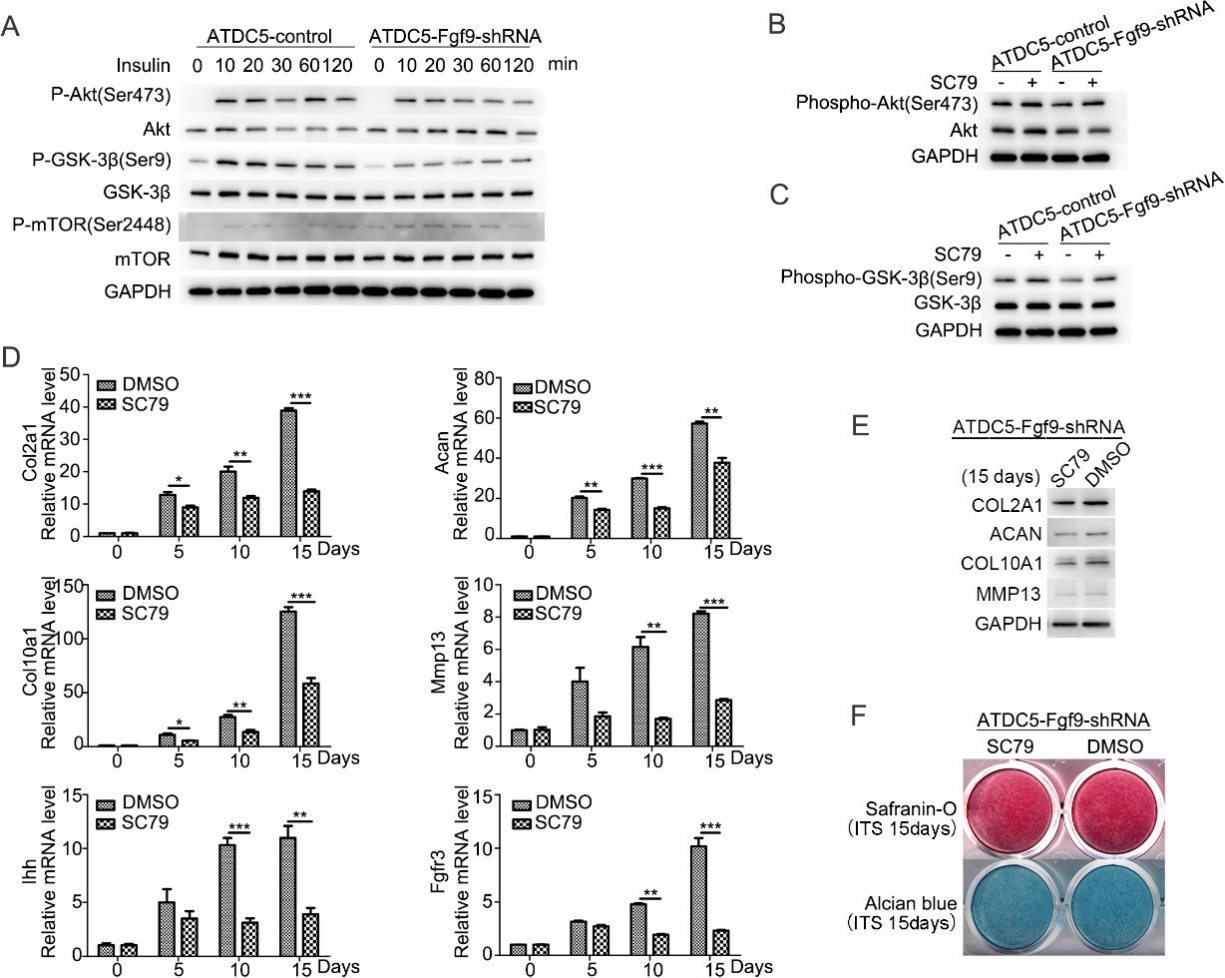
FGF9 silencing inhibits insulin-induced AKT/ GSK-3β pathway activation which participating in the differentiation of the ATDC5 cells. FGF9 silencing inhibits insulin-induced AKT and GSK-3β phosphorylation in the ATDC5 cells which was detected by Western blotting (A). Insulin-induced phosphorylation of AKT and GSK-3β in ATDC5-FGF9-shRNA cells is compensated by SC79 (B/C). When SC79 was added to the differentiation medium, the mRNA expression of chondrogenic markers Col2a1, Acan, Col10a1, Ihh, Mmp13 and FGFR3 were downregulated (D). The protein levels of Col2a1, Acan, Col10a1, Mmp13 were detected by Western blotting (E) on the induction day 15 with or without SC79, as well as cartilage matrix deposition by Safranin-O staining and Alcian blue staining (F). (* P<0.05; **P<0.01; ***P<0.005).

Previous studies have shown that AKT signaling plays an important regulatory role in chondrocytes differentiation [15, 16]. In order to discover the possible mechanism of FGF9 involved in insulin-induced chondrogenic differentiation of ATDC5 cells, the role of AKT signal was investigated. First, we examined AKT phosphorylation in ATDC5 cells induced by 10 μg/ml insulin within 2 hours (Fig 4A). AKT phosphorylation of ATDC5-FGF9-shRNA cells was downregulated compared to ATDC5 control cells. Next, we detected mTOR pathways and GSK-3β pathways, which are reported as regulated by AKT signaling [17]. Compared with ATDC5 control cells, FGF9 silencing downregulated insulin-induced phosphorylation of GSK-3β, but no significant change was shown in mTOR phosphorylation (Fig 4A).

Furthermore, 8 μM SC79 (an activator of AKT phosphorylation) was used during insulin-induced differentiation of ATDC5-FGF9-shRNA cells, and SC79 was found to increase the phosphorylation of GSK-3β after 30 minutes (Fig 4B and 4C). The mRNA expression was downregulated in chondrogenic genes Col2a1, Acan, Col10a1, Ihh, Mmp13 and FGFR3 (Fig 4D), which was consistent with the results of Western blotting, and Alcian blue and Safranin-O staining was attenuated (Fig 4F), indicating that SC79 inhibited the differentiation of ATDC5-FGF9-shRNA cells.

## Discussion

Early chondrocytes origin from mesenchymal precursor cells after cellular condensation [18]. This process, termed chondrogenesis, is characterized by producing chondrocyte-specific extracellular matrix (ECM) components, including COL2A1, and ACAN [18]. Subsequently, the number of chondrocytes increased and extracellular matrix was produced during the proliferative phase. The so-called then withdraw from the cell cycle and begin to differentiate into prehypertrophic chondrocytes [18]. In the stage of prehypertrophy, the expression of early cartilage matrix genes is still significant, while prehypertrophic specific markers such as IHH appear, and expression of hypertrophic markers, such as COL10A1, begins [19]. Following this is an expansion of cytoplasmic volume, prehypertrophic cells enter the stage of hypertrophic chondrocytes and release MMP13 to reshape the extracellular matrix [20]. These steps are precisely regulated by a variety of hormones, growth factors and cytokines, and their downstream intracellular signaling pathways and transcriptional regulation [1]. In the present study, we showed that FGF9, a member of FGFs, acted as a regulator of early chondrogenic differentiation partly by modulating the phosphorylation of AKT and GSK-3β.

Previous studies have shown that FGF9 is expressed in the proximity of developing skeletal elements of fetal mice [8]. Our in vivo experiment revealed the spatial expression of FGF9 in the growth cartilages of the forelimb, the posterior limb, the mandibular condyle and the cranial base from postnatal mice. Positive staining for FGF9 was observed in the both immature chondrocytes and mature chondrocytes in all these specimens. Furthermore, the expression of FGF9 in the hypertrophic chondrocytes was increased as differentiation from resting chondrocytes in the limbs and the cranial base. Consistently, an increased expression of FGF9 mRNA and protein levels was shown during the early differentiation of ATDC5 cells. Taken together, these findings appear to indicate that FGF9 may participate in early chondrogenic differentiation as well as in late stage of chondrogenesis during postnatal bone growth.

Our studies found that FGF9 silencing promoted proliferation of undifferentiated ATDC5 cells. However, early studies carried out anti-bromodeoxyuridine (BrdU) immunohistochemistry and showed that the proliferation of chondrogenic mesenchymal cells was not affected in FGF9-/- mice by E13.5, but a significant reduction was detected in proliferation of FGF9-/- femoral chondrocytes at E14.5 compared to control [8, 10]. The different results may be due to these experiments detected cell proliferation of different chondrogenic differentiation stages. Besides, FGF9 inhibited cell proliferation and apoptosis in remodeled pleura after adenoviral transduction in vivo [21]. This suggests that FGF9 may participate in the regulation of cell proliferation. The role of FGF9 in the regulation of proliferation of some other cells, especially cartilage-associated tumor cells, is postulated.

In humans and mice, FGFs phosphorylates and activates multiple intracellular signals and regulates a variety of cellular activities via binding to FGFR1-FGFR4 receptors, which are tyrosine kinase receptors on the surface of cell membranes [22]. FGF9, with the core structure of clover, can activate FGFR1/2 in the osteoprogenitor or FGFR3 in chondrocytes by binding with heparin [23]. FGFR3 is a negative regulator of postnatal bone growth, playing important role both in proliferating and prehypertrophic chondrocytes in the growth plate [23]. We found that FGF9 silencing promoted the expression of FGFR3 during chondrogenic differentiation in ATDC5 cells, which may lead to a decrease in the positive effects of chondrocytes proliferation and differentiation.

Tyrosine kinase-mediated signaling modules that have been confirmed include phosphoinositide 3 kinase (PI3K), mitogen-activated protein kinase (MAPK), protein kinase C (PKC) as well as signal transcription and activation factor 1 (STAT1) [22]. The PI3K-AKT pathway is a signaling cascade functioning in both chondrogenesis of articular cartilage [24, 25], which is essential for insulin-like growth factor I (IGF-1) induced differentiation of early chondrocytes from mesenchymal precursor cells [26]. AKT signaling can be activated in resting and proliferating chondrocytes [15]. Activation of AKT signaling promoted chondrocyte proliferation and inhibited hypertrophic differentiation during embryonic chondrogenesis [15]. Other studies showed that activating AKT signaling promoted the chondrogenic differentiation of ATDC5 cells to form nodules in the absence of insulin stimulation [16]. Previous studies have shown that FGF1, as an upstream signaling of AKT kinase, plays an important regulatory role in chondrocytes proliferation and differentiation [27]. In order to discover the possible mechanism of FGF9 involved in insulin-induced chondrogenic differentiation of ATDC5 cells, the phosphorylation of AKT signal was investigated. Interestingly, we found that FGF9 silencing inhibited the insulin-induced activation of AKT signaling and promoted the chondrogenic differentiation of ATDC5.

For further studies, we look for downstream signals after AKT activation. AKT regulates the activities of many molecules including GSK-3, TSC2, FoxOs, BAD, MDM2, Caspase 9, AS160, eNOS, m TOR and PRAS40 by phosphorylation [17]. Only AKT/mTOR, AKT/FoxO and AKT/GSK3 pathways have been reported to be involved in the regulation of endochondral osteogenesis in current studies [17]. Forkhead box O (FOXO) transcription factors function as homeostasis regulators to maintain tissue homeostasis over time and coordinate responses to environmental changes, including growth factor deprivation, metabolic stress (starvation) and oxidative stress [28]. PI3K/AKT/FoxO pathways may be involved in enhanced chondrogenic differentiation under hypoxia [29]. We examined the active of mTOR and GSK-3 to detect AKT downstream signaling pathways associated with FGF9 function during the early chondrogenic differentiation.

Mechanistic target of rapamycin (mTOR, also known as mammalian target of rapamycin) is a ubiquitous, central regulator of cell metabolism, proliferation and survival and critical for organismal growth and homeostasis [30, 31]. The AKT-mTOR pathway was responsible for positive regulation of all the four processes of chondrocyte differentiation, perhaps via regulating Ihh expression [17, 32]. Inhibition of mTOR signaling greatly impaired cartilage nodule formation in chondrogenic ATDC5 cells [33]. In our studies, the phosphorylation of mTOR was not obviously changed when enhancing chondrogenic differentiation with FGF9 silencing in ATDC5 cells.

Previous studies found that the AKT-GSK3 pathway negatively regulated early chondrocyte differentiation in mouse limb buds [17]. In our study, the phosphorylation level of GSK-3β decreased after 10 ug/ml insulin was added to ATDC5-FGF9-shRNA cells compared to the control. This suggests that FGF9 in chondrocyte can regulate the activity of GSK-3β. SC79 is a direct activator of AKT, which can enhance the phosphorylation level of AKT [34]. SC79 inhibits GSK-3β activity by activating AKT for treatment of early brain injury following subarachnoid hemorrhage [35]. When AKT activator SC79 was added to ATDC5-FGF9-shRNA cells, the phosphorylation and inactivation of GSK-3β upregulated significantly. At the same time, adding SC79 inhibited the early chondrogenic differentiation of ATDC5-FGF9-shRNA cells. It is indicated that FGF9 may inhibit the early chondrocyte differentiation, partly via the AKT/GSK-3β pathway.

Glycogen synthase kinase (GSK)-3 is a serine/threonine kinase that can be phosphorylated and inactivated by upstream kinases such as PI3K/AKT, cyclic AMP-dependent protein kinase (PKA), and protein kinase C (PKC) and regulating various cellular functions [36]. Studies have shown GSK-3β is involved in chondrocyte proliferation and differentiation, which can be regulated by multiple factors including cGMP-dependent protein kinase II (cGKII), bone morphogenetic protein-2 (BMP-2), parathyroid bormone-related protein (PTHrP), peroxisome proliferator activated receptor γ (PPARγ) and so on [37–41].

Interestingly, according to our experiment, the activity of GSK-3β increased after FGF9 silencing, which appeared earlier than the upregulation of AKT phosphorylation level. This implies that FGF9 can regulate the activity of GSK-3β through other signaling pathways during the early chondrogenic differentiation. Furthermore, GSK-3β has been reported to promote the degeneration of β-catinen in the process of chondrogenic differentiation and subsequently inactivate Wnt signaling [42, 43]. Wnt/β-catenin may be one of the downstream signals of GSK-3β kinase in the regulation of chondrogenic differentiation by FGF9. Early studies have shown that inhibition of GSK3 activity can significantly inhibit the proliferation and differentiation of chondrocytes, and the expression of FGF18 is inhibited [44]. In early embryonic development, FGF9 plays an important role in chondrocyte differentiation and its function is redundant with that of FGF18 [10]. In recent studies, we find that FGF9 can inhibit early chondrocyte differentiation through the AKT/GSK-3β pathway. It can be assumed that the regulation of FGF9 and FGF18 expression may be related to GSK3 signaling.

Intra-articular injection of exogenous FGF9 can delay the degradation of articular cartilage and aggravate osteophyte formation in post-traumatic osteoarthritis, but its mechanism is not clear [45]. GSK-3 is an important regulator of matrix metalloproteinase (MMP)-mediated destruction of articular cartilage, which inhibits catabolic activities by stimulating inflammation [46]. Therapeutic strategies to maintain the activity of GSK-3 in cartilage may help to reduce the abnormal activity of MMP during pathological joint destruction [46]. AKT/GSK-3β/β-catenin pathway, acting as the downstream of interleukin-1 receptor (type 1, IL-1R1)/MYD88 signaling, regulates the proliferation, migration and differentiation of mesenchymal stem cells during bone tissue injury [47]. It implies that GSK-3 is involved in the proliferation and differentiation of chondrocytes, as well as in the occurrence and development of osteochondral tissue damage and osteoarthritis. Our study shows that FGF9 can regulate the AKT/GSK-3β signaling pathway in chondrocytes. FGF9 may be involved in the occurrence and development of osteochondral diseases, and may become a new target for the treatment of osteoarthritis and other cartilage-related diseases.

## Conclusion

We revealed for the first time that FGF9 inhibited early chondrocyte differentiation, partly via AKT/GSK-3β pathway, which provided novel insights into the understanding and further study of the mechanism of FGFs in the early differentiation of chondrocyte and skeletal development, as well as new therapeutic approaches for cartilage-related disease.

## Supporting information

S1 Raw dataset (XLSX)

S1 Raw images (PDF)
