# Supplementary material for: Fibroblast Growth Factor 9 (FGF9) negatively regulates the early stage of chondrogenic differentiation
Source: PLoS One. 2021 Feb 2;16(2):e0241281. doi: 10.1371/journal.pone.0241281 (PMC7853451; doi:10.1371/journal.pone.0241281)
Supplement: S1 Raw images — (PDF) [file pone.0241281.s002.pdf]

Fig2.D

Western blot-Anti-FGF9 antibody (ab71395)

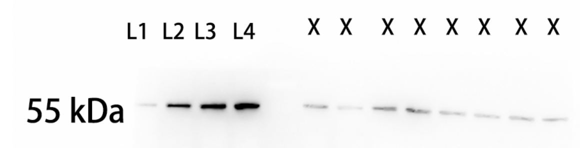

Observed band size：55 kDa

Western blot -GAPDH Antibody (AB0037)

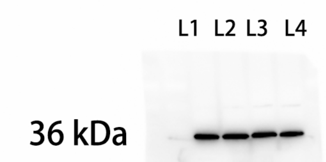

Lane 1 : ATDC5 cells induced to differentiate for 0 days
Lane 2: ATDC5 cells induced to differentiate for 5 days
Lane 3: ATDC5 cells induced to differentiate for 10 days
Lane 4: ATDC5 cells induced to differentiate for 15 days
Developed using the ECL technique

Fig3.C

Western blot-Anti-FGF9 antibody (AB71395)

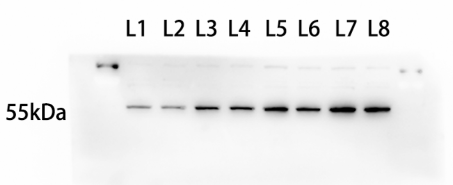

Western blot-GAPDH Antibody (AB0037)

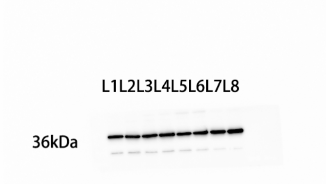

Lane 1 : ATDC5-control cells induced to differentiation for 0 days
Lane 2 : ATDC5-Fgf9-shRNA cells induced to differentiation for 0 days
Lane 3 : ATDC5-control cells induced to differentiation for 5 days
Lane 4 : ATDC5-Fgf9-shRNA cells induced to differentiation for 5 days
Lane 5 : ATDC5-control cells induced to differentiation for 10 days
Lane 6 : ATDC5-Fgf9-shRNA cells induced to differentiation for 10 days
Lane 7 : ATDC5-control cells induced to differentiation for 15 days
Lane 8 : ATDC5-Fgf9-shRNA cells induced to differentiation for 15 days

Developed using the ECL technique

Fig 4A

Western blot-Phospho-Akt (Ser473) (D9E) XP Rabbit mAb

Western blot-Akt (C67E7) Rabbit mAb

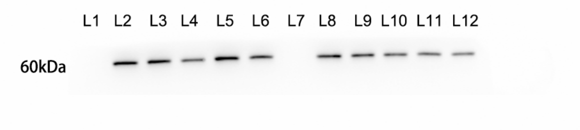

Western blot-Phospho-GSK-3 β (Ser9) (5B3) Rabbit mAb

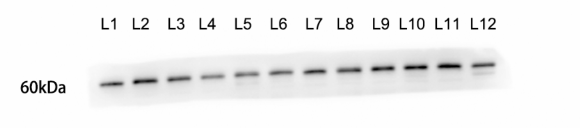

Western blot-GSK-3 β (D5C5Z) XP Rabbit mAb

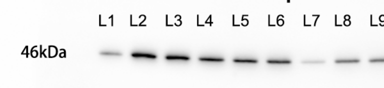

Western blot-Phospho-mTOR(Ser2448) (D9C2) XP Rabbit mAb

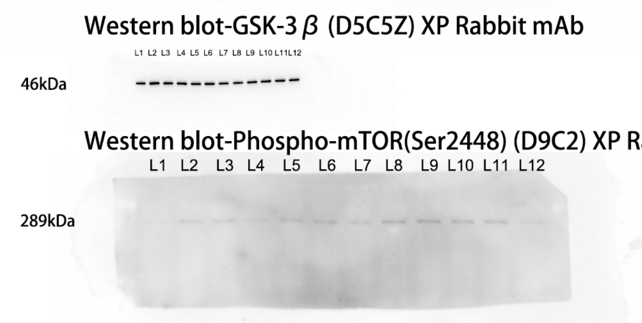

Western blot-mTOR (7C10) Rabbit mAb

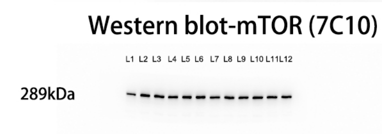

Western blot-GAPDH Antibody (AB0037)

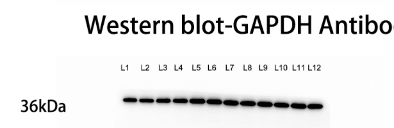

Lane 1: ATDC5-control cells induced by 10 μg/ml insulin for 0 min
Lane 2: ATDC5-control cells induced by 10 μg/ml insulin for 10 min
Lane 3: ATDC5-control cells induced by 10 μg/ml insulin for 20 min
Lane 4: ATDC5-control cells induced by 10 μg/ml insulin for 30 min
Lane 5: ATDC5-control cells induced by 10 μg/ml insulin for 60 min
Lane 6: ATDC5-control cells induced by 10 μg/ml insulin for 120 min
Lane 7: ATDC5-Fgf9-shRNA cells induced by 10 μg/ml insulin for 0 min
Lane 8: ATDC5-Fgf9-shRNA cells induced by 10 μg/ml insulin for 10 min
Lane 9: ATDC5-Fgf9-shRNA cells induced by 10 μg/ml insulin for 20 min
Lane 10: ATDC5-Fgf9-shRNA cells induced by 10 μg/ml insulin for 30 min
Lane 11: ATDC5-Fgf9-shRNA cells induced by 10 μg/ml insulin for 60 min
Lane 12: ATDC5-Fgf9-shRNA cells induced by 10 μg/ml insulin for 120 min

Fig 4B

Western blot-Phospho-Akt (Ser473) (D9E) XP Rabbit mAb

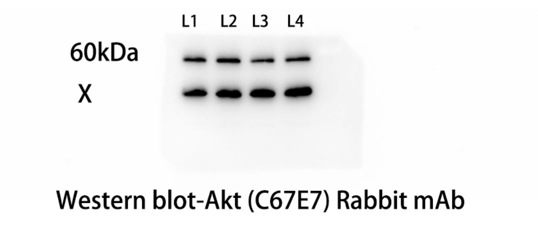

Western blot-Akt (C67E7) Rabbit mAb

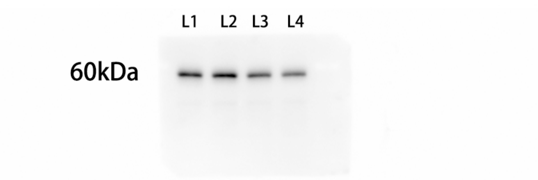

Western blot-GAPDH Antibody (AB0037)

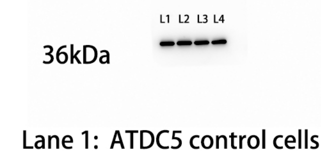

Lane 1: ATDC5 control cells treated with no SC79 for 30 min
Lane 1: ATDC5 control cells treated with SC79 for 30 min
Lane 1: ATDC5-Fgf9-shRNA cells treated with no SC79 for 30 min
Lane 1: ATDC5-Fgf9-shRNA cells treated with SC79 for 30 min

Fig 4C

Western blot-Phospho-GSK-3 β (Ser9) (5B3) Rabbit mAb

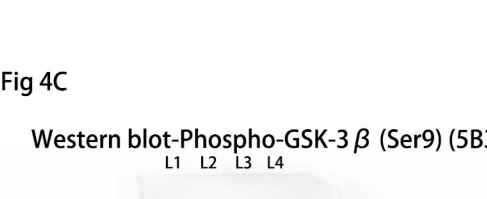

Western blot-GSK-3 β (D5C5Z) XP Rabbit mAb

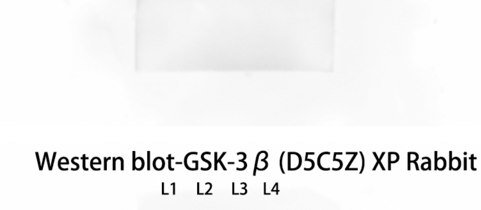

Western blot-GAPDH Antibody (AB0037)

Lane 1: ATDC5 control cells treated with no SC79 for 30 min
Lane 1: ATDC5 control cells treated with SC79 for 30 min
Lane 1: ATDC5-Fgf9-shRNA cells treated with no SC79 for 30 min
Lane 1: ATDC5-Fgf9-shRNA cells treated with SC79 for 30 min

Fig 2C/3I/4E

Western blot-Collagen II Polyclonal Antibody

Western blot-Collagen X Antibody

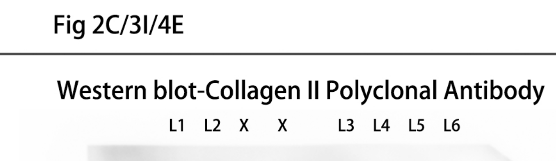

Western blot -GAPDH polyclonal antibody

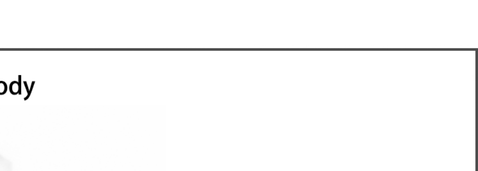

Western blot-Aggrecan Ab

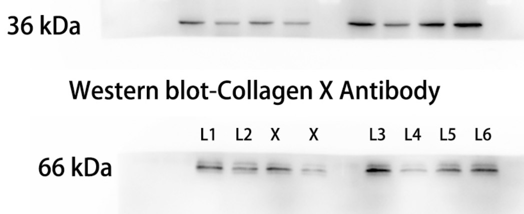

Western blot -GAPDH polyclonal antibody

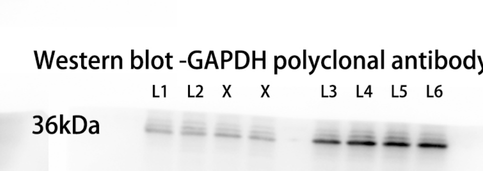

Western blot-MMP-13 polyclonal antibody

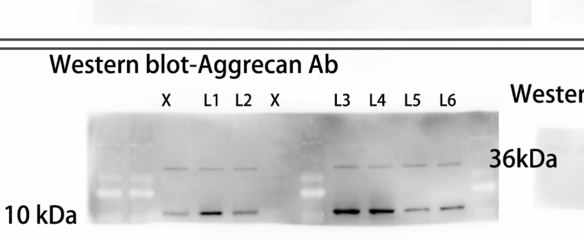

Western blot -GAPDH polyclonal antibody

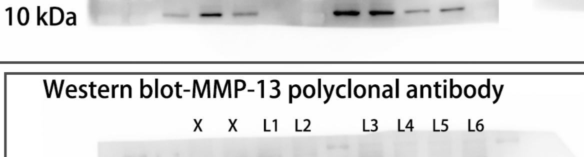

Lane 1: ATDC5 cells induced to differentiate for 15 days
Lane 2: ATDC5 cells induced to differentiate for 0 days
Lane 3: ATDC5-Fgf9-shRNA cells induced to differentiation for 15 days
Lane 4: ATDC5-control cells induced to differentiation for 15 days
Lane 5: ATDC5-Fgf9-shRNA cells treated with SC79 and induced to differentiation for 15 days
Lane 6: ATDC5-Fgf9-shRNA cells treated with no SC79 and induced to differentiation for 15 days
